# Trimethoprim use for urinary tract infection and risk of adverse outcomes in older patients: cohort study

**DOI:** 10.1136/bmj.k341

**Published:** 2018-02-09

**Authors:** Elizabeth Crellin, Kathryn E Mansfield, Clémence Leyrat, Dorothea Nitsch, Ian J Douglas, Adrian Root, Elizabeth Williamson, Liam Smeeth, Laurie A Tomlinson

**Affiliations:** 1 Department of Non-communicable Disease Epidemiology, London School of Hygiene and Tropical Medicine, Keppel Street, London WC1E 7HT, UK; 2 Department of Medical Statistics, Faculty of Epidemiology and Population Health, London School of Hygiene and Tropical Medicine, London, UK

## Abstract

**Objective:**

To determine if trimethoprim use for urinary tract infection (UTI) is associated with an increased risk of acute kidney injury, hyperkalaemia, or sudden death in the general population.

**Design:**

Cohort study.

**Setting:**

UK electronic primary care records from practices contributing to the Clinical Practice Research Datalink linked to the Hospital Episode Statistics database.

**Participants:**

Adults aged 65 and over with a prescription for trimethoprim, amoxicillin, cefalexin, ciprofloxacin, or nitrofurantoin prescribed up to three days after a primary care diagnosis of UTI between April 1997 and September 2015.

**Main outcome measures:**

The outcomes were acute kidney injury, hyperkalaemia, and death within 14 days of a UTI treated with antibiotics.

**Results:**

Among a cohort of 1 191 905 patients aged 65 and over, 178 238 individuals were identified with at least one UTI treated with antibiotics, comprising a total of 422 514 episodes of UTIs treated with antibiotics. The odds of acute kidney injury in the 14 days following antibiotic initiation were higher following trimethoprim (adjusted odds ratio 1.72, 95% confidence interval 1.31 to 2.24) and ciprofloxacin (1.48, 1.03 to 2.13) compared with amoxicillin. The odds of hyperkalaemia in the 14 days following antibiotic initiation were only higher following trimethoprim (2.27, 1.49 to 3.45) compared with amoxicillin. However, the odds of death within the 14 days following antibiotic initiation were not higher with trimethoprim than with amoxicillin: in the whole population the adjusted odds ratio was 0.90 (95% confidence interval 0.76 to 1.07) while among users of renin-angiotensin system blockers the odds of death within 14 days of antibiotic initiation was 1.12 (0.80 to 1.57). The results suggest that, for 1000 UTIs treated with antibiotics among people 65 and over, treatment with trimethoprim instead of amoxicillin would result in one to two additional cases of hyperkalaemia and two admissions with acute kidney injury, regardless of renin-angiotensin system blockade. However, for people taking renin-angiotensin system blockers and spironolactone treatment with trimethoprim instead of amoxicillin there were 18 additional cases of hyperkalaemia and 11 admissions with acute kidney injury.

**Conclusion:**

Trimethoprim is associated with a greater risk of acute kidney injury and hyperkalaemia compared with other antibiotics used to treat UTIs, but not a greater risk of death. The relative risk increase is similar across population groups, but the higher baseline risk among those taking renin-angiotensin system blockers and potassium-sparing diuretics translates into higher absolute risks of acute kidney injury and hyperkalaemia in these groups.

## Introduction

Co-trimoxazole is a combination antibiotic drug containing trimethoprim and sulfamethoxazole, prescribed for multiple indications and is the fourth most commonly prescribed antibiotic in the USA.^1^ Its use has been associated with an increased risk of sudden death among people taking renin-angiotensin system blockers.^2^ ^3^ This may be owing to acute kidney injury, a rapid deterioration in kidney function.^4^ Alternately, co-trimoxazole and trimethoprim alone have both been associated with an increased risk of an acute rise in potassium levels, hyperkalaemia, which can cause fatal cardiac arrhythmias.^3^ ^5^ ^6^ ^7^ ^8^

Existing evidence has important limitations. It is not clear if the association between co-trimoxazole and adverse outcomes is owing to the sulfamethoxazole or the trimethoprim component. The observed association may be owing to confounding if the combination antibiotic was used for patients with more severe infections than the antibiotics it was compared with. Finally, existing evidence is primarily associated with specific groups of patients such as those taking renin-angiotensin system blockers. In the UK, co-trimoxazole is licensed for specific, uncommon indications and trimethoprim is more commonly used. However, there are efforts to reduce trimethoprim prescribing due to increasing antimicrobial resistance.^9^ In 2015 over 3.7 million prescriptions for trimethoprim were dispensed in England and it remains a first line option for treatment of uncomplicated urinary tract infections (UTIs).^10^ Despite this, whether trimethoprim alone is linked to sudden death both in the general population and in high risk groups is unknown.

Our study therefore aimed to investigate the association between trimethoprim and acute kidney injury, hyperkalaemia, or sudden death in a cohort of patients aged 65 and over. To limit confounding by antibiotic indication we restricted our analysis to patients with an antibiotic prescription for the same indication (UTI) and examined the risk of adverse outcomes in patients prescribed trimethoprim and four comparison antibiotics (amoxicillin, cefalexin, ciprofloxacin, and nitrofurantoin). However, even when treatment is restricted to the same indication, different classes of antibiotic drugs are prescribed in slightly different clinical scenarios. For simple UTIs in adults, current UK guidelines recommend nitrofurantoin as the first line treatment (except for patients with poor renal function) and trimethoprim as a first line alternative where there is low risk of microbial resistance.^11^ Ciprofloxacin and cefalexin are not currently recommended treatments for simple UTIs (although ciprofloxacin is a first line option for pyelonephritis) but are used for patients with resistance to first line antibiotics. In addition, ciprofloxacin and cefalexin were used in practice as treatment for simple UTIs during the years covered by this study.^12^ We aimed to address the impact of these different usage patterns on the outcomes through adjustment for a range of comorbidities and sensitivity analyses.

## Methods

### Study design and setting

We undertook a cohort study using electronic clinical records from adults attending primary care practices contributing to the UK Clinical Practice Research Datalink (CPRD GOLD) and linked hospital record data from the Hospital Episode Statistics (HES) database. CPRD is a database of primary care electronic health record data from participating general practices, covering 7% of the UK population.^13^ Included patients are largely representative of the UK population in terms of age, sex, ethnicity, and body mass index.^13^ ^14^ ^15^ HES records cover all admissions for National Health Service (NHS) funded patients treated in either English NHS trusts or by independent providers.^16^ Seventy-five percent of English general practices included in CPRD are linked to HES data.^13^ The study period was from 1 April 1997 to 30 September 2015 (the period covered by HES data linkage to CPRD).

### Participants, exposures, and outcomes

We identified all adults aged 65 years and over during the study period (April 1997 to September 2015). We chose an older population as this is a clinically important group at high risk of adverse health outcomes. In addition, they are more likely to be well monitored, enabling reliable recording of comorbidities and quantification of baseline renal function.

Individuals were eligible for the study by receiving a prescription for an antibiotic for a urinary tract infection (UTI) after the latest of the following: 65th birthday; date practice reached CPRD quality control standards (to ensure data quality); or one year after practice registration date (to ensure that we had reliable measures of incident morbidity). Patients were no longer eligible to be included from the earliest of the following: date of death; left practice; or last data collection from practice. We excluded patients who developed end stage renal disease before they were eligible for inclusion.^17^

#### Exposures

The date of inclusion was the day of initiation for any of five antibiotic drugs (trimethoprim, amoxicillin, cefalexin, ciprofloxacin, and nitrofurantoin) recorded up to three days after a primary care morbidity code for uncomplicated UTI (ie, excluding more severe conditions such as pyelonephritis). We allowed a gap of three days between UTI diagnosis and treatment with an antibiotic to allow for delays between microbiological diagnosis and treatment. To ensure reliable measures of antibiotic exposure, we excluded any UTI episodes treated with antibiotics where two or more of the study antibiotics were prescribed on the same day. We excluded prescriptions for co-trimoxazole and did not include patients treated with co-amoxiclav in the amoxicillin comparison group as in the UK these drugs are prescribed for more severe or atypical UTIs. We also excluded any UTI episodes where a patient received one or more of the five study antibiotics in the 14 days before the UTI record to ensure that we were identifying the first consultation for an episode of UTI. Finally, we excluded any UTI episodes where a code for a non-UTI infection was recorded in the three days before antibiotic prescription.

#### Outcomes

We investigated the outcomes acute kidney injury, hyperkalaemia, and death recorded within 14 days of antibiotic initiation for UTI. Acute kidney injury was defined as hospital admission with acute kidney injury using ICD-10 (international classification of diseases, 10th revision) codes recorded in any diagnostic position of any inpatient episode starting within 14 days of antibiotic initiation.^17^ ^18^ Hyperkalaemia was identified in the 14 days after antibiotic initiation using morbidity coding in primary (Read codes) or secondary (ICD-10 codes) care, or a potassium level of 6 mmol/L or more recorded in primary care. Death was identified as the earliest record of death from Read codes in CPRD, CPRD defined death date, ICD-10 codes in HES, and the Office for National Statistics date of death.

All morbidity code lists are available to download,^19^ and were either developed for use in other studies, or were developed in a consensus procedure by two authors with clinical experience in the NHS.

### Covariates

Based on a priori knowledge, we considered the following variables as potential confounders of the relation between trimethoprim and acute kidney injury, hyperkalaemia, or sudden death: sex, age, calendar period, chronic comorbidities, history of renal or urological disease, baseline renal function, prescription for renin-angiotensin system blockers (including angiotensin converting enzyme inhibitors and angiotensin receptor blockers) or potassium-sparing diuretics, lifestyle factors (smoking, alcohol intake, and body mass index), ethnicity, and socioeconomic status. All covariates other than sex and ethnicity were updated over time.

Age group was defined in the following bands at time of antibiotic initiation: 65–69; 70–74; 75–79; 80–84; and 85 and over.

We included calendar period (1997–2000, 2001–04, 2005–08, 2009–11, and 2012–15) as a covariate to adjust for the many changes in clinical, diagnostic, and administrative practices over the study period that may influence the choice of antibiotic treatment for UTI, the measurement of renal function, and registration of outcomes.

Chronic comorbidities included as confounders were diabetes mellitus, ischaemic heart disease, cardiac failure, arrhythmia, and hypertension, identified from both primary care and hospital data. Individuals were considered to have a specific comorbidity if they had a code recorded in their electronic health records before a UTI episode treated with antibiotics.

Baseline renal function was defined using the most recent biochemical test results recorded in primary care at any time before each UTI treated with antibiotics. We used serum creatinine test results to calculate estimated glomerular filtration rate using the Chronic Kidney Disease Epidemiology Collaboration (CKD-EPI) equation.^20^ We categorised estimated glomerular filtration rate into the following bands analogous to chronic kidney disease stages: 60 mL/min/1.73m^2^ and over; 45–59 mL/min/1.73m^2^; 30–44 mL/min/1.73m^2^; and less than 30 mL/min/1.73m^2^. This created possible selection bias since those at greater risk of renal disease are more likely to have renal function measured. Therefore, we included an absent renal function category for those with no recorded serum creatinine test result before UTI treated with antibiotics.

History of renal and urological disease were identified using primary care records and classified in the following categories: prostatic hypertrophy, renal calculi, urological malignancies, and renal structural anomalies. To identify historic diagnoses that may influence prescribing rather than a more immediate condition that may have caused the infection (and therefore potentially be on the causal pathway) we identified renal disease based on codes recorded more than a year before each UTI episode treated with antibiotics.

Exposure to renin-angiotensin system blockers or potassium-sparing diuretics was defined using prescription data as a current prescription at the time of a UTI treated with antibiotics and categorised as neither a renin-angiotensin system blocker nor a potassium-sparing diuretic, either a renin-angiotensin system blocker or a potassium-sparing diuretic, or renin-angiotensin system blockers in combination with potassium-sparing diuretics. We calculated prescription duration using the quantity of medication prescribed and daily dose recorded; when these data were not available, we assumed the population median prescription duration of 28 days. We assumed exposure to medications started on the date of the prescription. We constructed continuous courses of therapy by allowing for a gap of 60 days between consecutive prescriptions. We therefore defined a current prescription when a UTI episode treated with antibiotics occurred during a continuous course of drug therapy.

We used existing morbidity code lists and algorithms for ethnicity,^14^ smoking status, alcohol intake, and body mass index.^15^ A large proportion of ethnicity data were missing so we only adjusted for ethnicity as a covariate in sensitivity analyses.^14^ Lifestyle covariates (smoking, alcohol intake, and body mass index) were defined using the closest primary care record to the date of antibiotic prescription. Socioeconomic status was defined using general practice level quintiles of index of multiple deprivation scores. The effects of lifestyle covariates and socioeconomic status were thought to be largely captured by comorbidity data; we therefore only adjusted for these covariates in sensitivity analyses.

### Statistical analysis

We calculated odds ratios for each outcome (acute kidney injury, hyperkalaemia, and death) within 14 days of antibiotic initiation for a UTI comparing each antibiotic drug (trimethoprim, cefalexin, ciprofloxacin, and nitrofurantoin) to amoxicillin (as the reference category) adjusting for potential confounders using logistic regression. We used robust standard errors to account for clustering by general practice. Separately, we repeated the analyses using robust standard errors to account for clustering by patient to account for some patients contributing multiple UTI episodes to the analysis. We initially adjusted for sex and age only, and then fitted an adjusted model using sex, age, calendar period, chronic comorbidities, baseline renal function, history of renal or urological disease, and use of renin-angiotensin system blockers and potassium-sparing diuretics.

### Sensitivity analyses

We tested the impact of our antibiotic exposure definition by repeating the main analysis after: defining exposure as antibiotic prescription on the same day as a record of a UTI morbidity code; excluding prescriptions for antibiotics where the patient received an antibiotic in the 28 days before index infection; and limiting the analysis to the first recorded UTI treated with antibiotics for each individual during eligible follow-up. We then tested the impact of defining more immediate outcomes by repeating the main analysis with all three outcomes defined within seven days (rather than 14 days) of index antibiotic initiation. We also repeated the main analysis additionally adjusting for lifestyle factors (smoking, alcohol intake, and body mass index) and socioeconomic status. We repeated the main analysis limiting to individuals who had ethnicity recorded in Clinical Practice Research Datalink (CPRD) or Hospital Episode Statistics (HES), and became eligible for study entry from 2006 when recording of ethnicity was rewarded in primary care leading to improvements in CPRD data completeness.^14^ We included ethnicity both in the equation used to calculate estimated glomerular filtration rate and as covariate in the analysis. Next, to more closely replicate previous studies,^2^ ^3^ ^5^ ^21^ we repeated the main analysis with the exposure defined as antibiotic prescription for any indication, and, separately, limiting to individuals who had a current prescription for a renin-angiotensin system blocker at the time of UTI treated with antibiotics examining death both at seven and 14 days. Finally , to ensure that we were comparing similar groups (to reduce confounding by indication), we examined the risks of all three outcomes after propensity score weighting (inverse probability of treatment weighting) of trimethoprim and amoxicillin users (full details in web appendix 1). In inverse probability of treatment weighting, patients are reweighted according to the inverse of their probability of receiving the treatment they actually received. The strength of inverse probability of treatment weighting compared with propensity score matching is that every patient is included in the analysis, whereas propensity score matching may lead to the exclusion of patients for which a good match cannot be found, therefore threatening the generalisability of the results.

All data management and analyses were performed using Stata version 14 (StataCorp, Texas, USA).

### Patient involvement

No patients were involved in setting the research question or the outcome measures, nor were they involved in developing plans for design or implementation of the study. No patients were asked to advise on interpretation or writing up of results. We are not able to disseminate the results of the research directly to study participants because the data used were anonymised. 

## Results

### Study population

**Fig 1:**
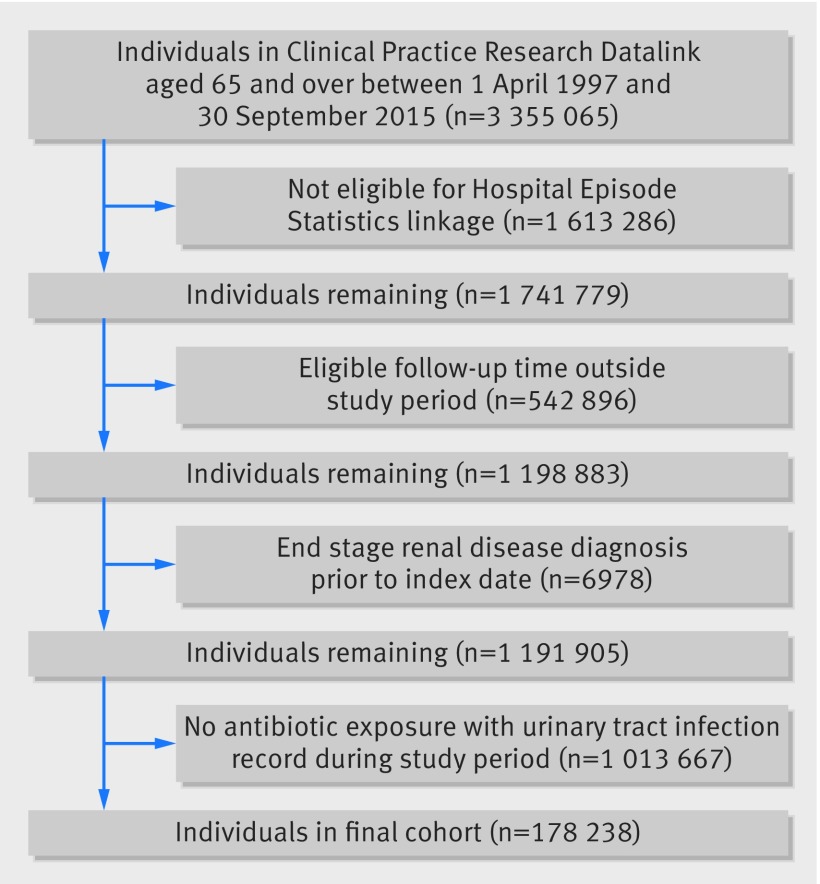
Flow diagram

Figure 1 shows that among a cohort of 1 191 905 patients aged 65 and over we identified 178 238 individuals with a least one urinary tract infection (UTI) treated with antibiotics, comprising a total of 422 514 episodes. Patients contributed a median of three UTIs treated with antibiotics (interquartile range 2–7) during a mean follow-up period of 9.1 (SD 5.0) years. Table 1 shows that of these UTIs, 5% (n=22 543) were treated with amoxicillin, 59% (251 193) with trimethoprim, 15% (64 885) with cefalexin, 5% (21 946) with ciprofloxacin, and 15% (61 947) with nitrofurantoin. There were a total of 1345 episodes of acute kidney injury, 648 episodes of hyperkalaemia, and 2214 deaths within 14 days of antibiotic initiation for a UTI. The majority of cases of hyperkalaemia were identified from primary care biochemical test results; 71% of all hyperkalaemia records within 14 days of a UTI treated with antibiotics were identified from test results, 18% were recorded using Read codes in primary care, and 11% from hospital records.

**Table 1:** Characteristics of the study population at time of antibiotic initiation for urinary tract infection for the whole study population and stratified by antibiotic drug. Values are numbers (percentages) unless stated otherwise

Characteristic	All*	Amoxicillin (Reference)	Trimethoprim	Cefalexin	Ciprofloxacin	Nitrofurantoin
Patients	178 238	17 536	135 665	38 425	15 594	39 200
UTIs	422 514	22 543	251 193	64 885	21 946	61 947
Median (IQR) UTIs per patient	3 (2-7)	4 (2-7)	3 (1-6)	4 (2-8)	4 (2-9)	4 (2-8)
Outcomes:						
Acute kidney injury	1345 (0.3)	66 (0.3)	914 (0.4)	120 (0.2)	83 (0.4)	162 (0.3)
Hyperkalaemia	648 (0.2)	25 (0.1)	463 (0.2)	68 (0.1)	28 (0.1)	64 (0.1)
Deaths	2214 (0.5)	154 (0.7)	1293 (0.5)	388 (0.6)	137 (0.6)	242 (0.4)
Female	333 466 (78.9)	16 957 (75.2)	201 043 (80.0)	51 147 (78.8)	13 591 (61.9)	50 728 (81.9)
Age:						
65-69	84 951 (20.1)	3767 (16.7)	51 943 (20.7)	12 382 (19.1)	4139 (18.9)	12 720 (20.5)
70-74	86 626 (20.5)	4289 (19.0)	52 049 (20.7)	12 926 (19.9)	4462 (20.3)	12 900 (20.8)
75-79	89 586 (21.2)	4823 (21.4)	52 913 (21.1)	14 132 (21.8)	4754 (21.7)	12 964 (20.9)
80-84	76 657 (18.1)	4436 (19.7)	44 577 (17.7)	12 308 (19.0)	4232 (19.3)	11 104 (17.9)
85+	84 694 (20.0)	5228 (23.2)	49 711 (19.8)	13 137 (20.2)	4359 (19.9)	12 259 (19.8)
Calendar period:						
1997-2000	49 032 (11.6)	2879 (12.8)	32 384 (12.9)	8540 (13.2)	2885 (13.1)	2344 (3.8)
2001-04	84 728 (20.1)	4798 (21.3)	52 464 (20.9)	16 890 (26.0)	5084 (23.2)	5492 (8.9)
2005-08	108 414 (25.7)	5463 (24.2)	61 632 (24.5)	24 701 (38.1)	6901 (31.4)	9717 (15.7)
2009-11	87 765 (20.8)	4666 (20.7)	51 744 (20.6)	9469 (14.6)	3926 (17.9)	17 960 (29.0)
2012-15	92 575 (21.9)	4737 (21.0)	52 969 (21.1)	5285 (8.1)	3150 (14.4)	26 434 (42.7)
Chronic comorbidities:						
Diabetes mellitus	86 321 (20.4)	4934 (21.9)	48 529 (19.3)	14 241 (21.9)	4955 (22.6)	13 662 (22.1)
Ischaemic heart disease	138 555 (32.8)	7942 (35.2)	79 547 (31.7)	22 368 (34.5)	7604 (34.6)	21 094 (34.1)
Cardiac failure	43 989 (10.4)	3016 (13.4)	24 333 (9.7)	10 226 (15.8)	2681 (12.2)	6221 (10.0)
Arrhythmia	64 500 (15.3)	4157 (18.4)	35 898 (14.3)	10 226 (15.8)	3596 (16.4)	10 623 (17.1)
Hypertension	255 759 (60.5)	14 017 (62.2)	149 252 (59.4)	38 675 (59.6)	13 165 (60.0)	40 650 (65.6)
Baseline renal function (mL/min/1.73m^2^):						
eGFR >=60	185 509 (43.9)	9118 (40.4)	108 806 (43.3)	23 929 (36.9)	8772 (40.0)	34 884 (56.3)
eGFR 45-59	97 201 (23.0)	5193 (23.0)	56 938 (22.7)	15 679 (24.2)	5215 (23.8)	14 176 (22.9)
eGFR 30-44	46 890 (11.1)	2868 (12.7)	26 442 (10.5)	8455 (13.0)	2801 (12.8)	6324 (10.2)
eGFR <30	13 566 (3.2)	1169 (5.2)	6848 (2.7)	2708 (4.2)	1060 (4.8)	1781 (2.9)
Absent	79 348 (18.8)	4195 (18.6)	52 159 (20.8)	14 114 (21.8)	4098 (18.7)	4782 (7.7)
History of renal or urological disease:						
Prostatic hypertrophy	21 528 (5.1)	1305 (5.8)	11 861 (4.7)	3447 (5.3)	2107 (9.6)	2808 (4.5)
Renal calculi	9087 (2.2)	568 (2.5)	4597 (1.8)	1621 (2.5)	788 (3.6)	1513 (2.4)
Malignancy	620 (0.1)	46 (0.2)	334 (0.1)	102 (0.2)	45 (0.2)	93 (0.2)
Structural abnormalities	4038 (1.0)	277 (1.2)	2021 (0.8)	712 (1.1)	307 (1.4)	721 (1.2)
Exposure to RAS blocker or KSD:						
Neither	272 610 (64.5)	14 338 (63.6)	164 932 (65.7)	41 882 (64.5)	13 809 (62.9)	37 649 (60.8)
One	143 682 (34.0)	7787 (34.5)	82 821 (33.0)	21 950 (33.8)	7757 (35.3)	23 367 (37.7)
Both	6222 (1.5)	418 (1.9)	3440 (1.4)	1053 (1.6)	380 (1.7)	931 (1.5)

UTI=urinary tract infection; IQR=interquartile range; eGFR=estimated glomerular filtration rate; RAS=renin-angiotensin system; KSD=potassium-sparing diuretic. *Individuals can contribute data from more than one urinary tract infection treated with antibiotics and can therefore be exposed to more than one class of antibiotic or have more than one urinary tract infection treated with the same antibiotic. Therefore, numbers exposed to each class of antibiotic do not total the whole cohort, as individuals may be included in more than one column

Table 1 shows the characteristics of patients at the time of antibiotic prescription for a UTI for the overall study population, and stratified by class of antibiotic prescribed. Amoxicillin or ciprofloxacin were more commonly used to treat UTIs in men and a slightly higher percentage of those prescribed amoxicillin were aged 85 and over. While the proportion of chronic comorbidities were broadly similar across the antibiotics, the patients prescribed trimethoprim had fewer comorbidities compared with amoxicillin. Individuals prescribed nitrofurantoin had better renal function (56% of UTIs treated with nitrofurantoin were in individuals with estimated glomerular filtration rate ≥60 mL/min/1.73m^2^, compared with 37% to 43% for other antibiotics). Ciprofloxacin was more likely to be prescribed for UTIs in patients with a history of prostatic hypertrophy or renal calculi (10% of patients prescribed ciprofloxacin had a history of prostatic hypertrophy compared with 5% to 6% for the other antibiotics, while 4% prescribed ciprofloxacin had a history of renal calculi compared with 2% to 3% for the other antibiotics).

### Association of trimethoprim with acute kidney injury, hyperkalaemia, or death

**Fig 2:**
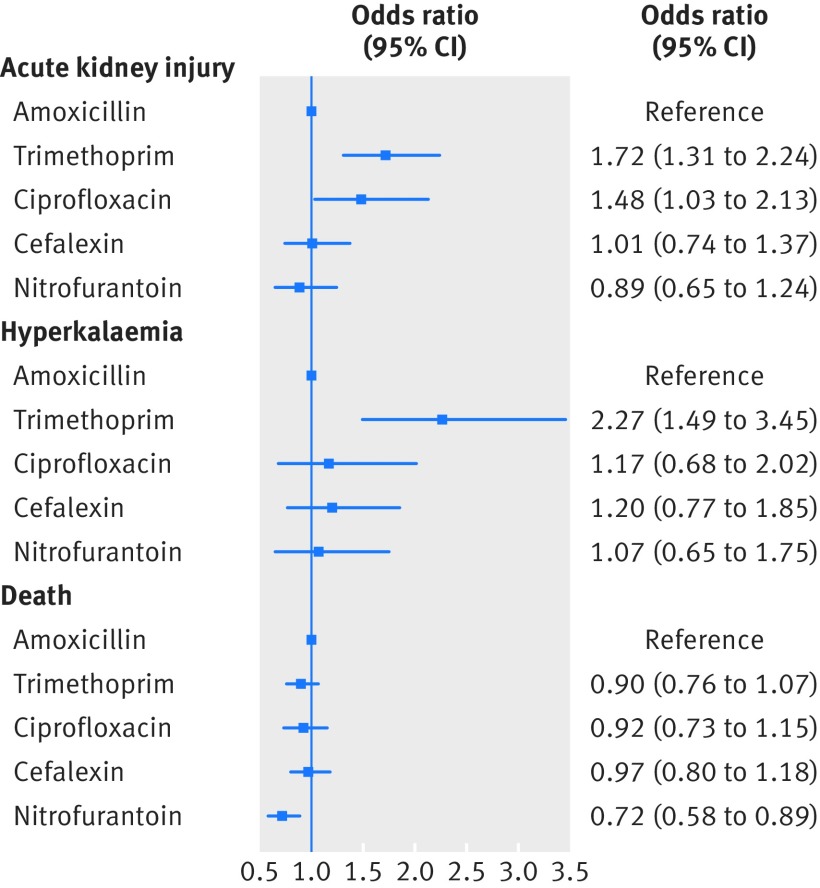
Odds ratios (95% confidence intervals) comparing the odds of acute kidney injury, hyperkalaemia, and death in the 14 days following initiation of different antibiotic drugs for treatment of urinary tract infection

Figure 2 shows the association between antibiotic prescription and all three adverse outcomes. In the 14 days after antibiotic initiation for a UTI, trimethoprim is associated with the highest odds of acute kidney injury (adjusted odds ratio 1.72, 95% confidence interval 1.31 to 2.24) and hyperkalaemia (2.27, 1.49 to 3.45) of all the antibiotics investigated. Ciprofloxacin was also associated with an increased odds of acute kidney injury (1.48, 1.03 to 2.13) but not of hyperkalaemia (1.17, 0.68 to 2.02). Cefalexin and nitrofurantoin were not associated with an increased odds of acute kidney injury or hyperkalaemia compared with amoxicillin. The odds of death within 14 days of antibiotic initiation for UTI were similar to amoxicillin for trimethoprim (0.90, 0.76 to 1.07) and the other antibiotics.

### Sensitivity analyses

We saw minimal differences in the odds ratios for acute kidney injury, hyperkalaemia, or sudden death comparing trimethoprim, cefalexin, ciprofloxacin, or nitrofurantoin with amoxicillin after the majority of sensitivity analyses, or after additionally adjusting for lifestyle factors, ethnicity, and socioeconomic status (web appendix 1). Repeating the main analysis excluding patients without known baseline renal function made minimal differences to the results (web appendix 1). Redefining exposure as antibiotic prescription for any indication (rather than only for a UTI) increased the observed effect size of the association between trimethoprim and acute kidney injury: the odds ratio comparing trimethoprim with amoxicillin increased from 1.72 to 2.36 (95% confidence interval 2.22 to 2.51). There were minimal changes in the sizes of the association with hyperkalaemia and death. When the analysis was restricted to users of renin-angiotensin system blockers at the time of a UTI (as opposed to adjusting for these drugs as covariates), the risk of acute kidney injury and hyperkalaemia after trimethoprim use were similar to the main analysis; adjusted odds ratio of 1.92 (1.29 to 2.87) for acute kidney injury and 2.22 (1.34 to 3.68) for hyperkalaemia. While the point estimate for death comparing trimethoprim with amoxicillin among users of renin-angiotensin system blockers was higher than among the whole population, confidence intervals were wide; within 14 days adjusted odds ratio for death was 1.12 (0.80 to 1.57). To enable comparison with other studies we counted the number of people prescribed renin-angiotensin system blockers who died with codes specifically suggestive of sudden death (I46, R96, R98, and R99) in the 14 days after antibiotic initiation. However, this included only six people so we were unable to analyse this outcome.

Finally, analyses using multivariable regression and inverse probability treatment weighting approaches comparing trimethoprim with amoxicillin users (prescribed for a UTI) were consistent with those from the main analysis (web appendix 1).

## Discussion

Our results show that in older patients in the general population, regardless of use of renin-angiotensin system blockers or potassium-sparing diuretics, treatment with trimethoprim for a urinary tract infection (UTI) is associated with a 72% increase in the odds of acute kidney injury and a greater than doubling of the odds of hyperkalaemia compared with amoxicillin. We also show that ciprofloxacin is associated with a 48% increase in the odds of acute kidney injury compared with amoxicillin. In contrast, no antibiotic was associated with increased risk of death within 14 days compared with amoxicillin. The relative risks of acute kidney injury, hyperkalaemia, and death were similar in the general population and among those prescribed renin-angiotensin system blockers after trimethoprim use for a UTI.

### Strengths and weaknesses of this study

This is the first study to quantify the association of trimethoprim with these outcomes, for an unselected general population cohort after a UTI. Our study used a large number of routine, prospectively collected clinical records from a UK general practice database that is broadly representative of the UK population.^13^ Our results are therefore generalisable to all patients aged 65 and over, in contrast to previous research restricted to select populations (eg, patients taking renin-angiotensin system blockers and potassium-sparing diuretics).^2^ ^3^ ^21^ We have investigated the risk of adverse outcomes after taking antibiotics prescribed for a UTI, thereby reducing the confounding by indication that has limited previous research.^2^ ^3^ ^4^ ^5^ ^21^ As we investigated the effect of trimethoprim alone, we can be clear about the likely causative agent compared with research on the combination product co-trimoxazole.

However, there are some important limitations. While we attempted to capture only simple UTIs (defined using primary care morbidity coding, but not excluding those with a history of more complex urological pathology) in our main analysis, we may have included patients with underlying urinary tract disorders, or other infections. Since different classes of antibiotic drugs are prescribed for different clinical scenarios, some degree of confounding by indication is unavoidable. However, we were able to reduce confounding by robustly defining and adjusting for variables that may have influenced the choice of antibiotic drug prescribed such as baseline renal function. As trimethoprim was less frequently prescribed for patients with urological pathology, this would likely have led to underestimating the odds of adverse outcomes, particularly acute kidney injury, for trimethoprim compared with the true result. Similarly, clinicians may have been cautious in prescribing trimethoprim to those at highest risk of acute kidney injury and hyperkalaemia, again leading to an underestimation of the true risk of adverse outcomes, particularly for those taking renin-angiotensin system blockers. However, the strongest evidence of adverse outcomes in association with trimethoprim use for those taking renin-angiotensin system blockers was only published towards the end of the period of this study.^2^ ^21^

Our assessment of antibiotic exposure was based on prescriptions alone and patients may not have collected or taken their medicine. This may have led to differential misclassification owing to the severity of the infection, with resulting over or under estimation of the true effect size. However, we have attempted to mitigate for this by limiting the study to simple UTIs and adjusting, in particular, for history of renal or urological disease.

We may also have misclassified the outcomes. Trimethoprim reduces tubular secretion of creatinine causing apparent renal impairment, although glomerular filtration rate does not fall.^22^ Lack of awareness of this physiological effect may have led clinicians to incorrectly diagnose acute kidney injury among the trimethoprim treated group, particularly given the current focus on creatinine based definitions of acute kidney injury. However, our definition of acute kidney injury relied on clinical coding of hospital admissions. In general, this leads to under ascertainment compared with analyses of serial creatinine tests but disproportionately captures more severe acute kidney injury.^23^ In addition, the increased risk of acute kidney injury dated back to 2001-04, before creatinine based definitions of acute kidney injury were in common use. It is also possible that there was a bias towards testing for or recording acute kidney injury or hyperkalaemia among patients taking trimethoprim if clinicians were aware of a potential association which would have led to an overestimation of the true risk of adverse outcomes.

### Results in context

Multiple historic case reports and case series have implicated co-trimoxazole as a cause of reduced renal function,^8^ ^24^ interstitial nephritis,^25^ ^26^ or acute tubular necrosis.^27^ Among patients receiving co-trimoxazole in hospital, 6% developed a creatinine based definition of acute kidney injury that was attributed to the antibiotics.^4^ However, we believe our study to be the first to show and quantify an association between trimethoprim alone and acute kidney injury in the general population. This is an important distinction as the sulphonamide antibiotics (including sulfamethoxazole) have been long recognised to be associated with a substantial risk of acute renal impairment, which could have been assumed to be causal.^28^ Our finding of an association of ciprofloxacin with acute kidney injury is similar to another population based study that showed that users of fluoroquinolones had a 2.18-fold (95% confidence interval 1.74 to 2.73) higher rate of acute kidney injury.^29^ However, ciprofloxacin is recommended for treatment of pyelonephritis, so an association with acute kidney injury may be due to confounding by severity of infection.

An association between both co-trimoxazole, or trimethoprim alone, with hyperkalaemia is well reported, particularly in association with renin-angiotensin system blockers.^3^ ^7^ ^8^ ^30^ ^31^ ^32^ ^33^ Our results suggest that, regardless of other drug use (renin-angiotensin system blockers or potassium-sparing diuretics), trimethoprim (but not ciprofloxacin), is associated with a more than doubling of the odds of hyperkalaemia. There is an additional increase in the odds of hyperkalaemia after a UTI for those prescribed renin-angiotensin system blockers, and greater than sixfold increase in association with concomitant use of a potassium-sparing diuretic, regardless of antibiotic choice. Our findings are in keeping with those of a Canadian nested case-control study of older patients taking renin-angiotensin system blockers that identified a nearly sevenfold increased risk of hospital admission for hyperkalaemia with co-trimoxazole compared with other antibiotic drugs.^3^

This cohort covers the years 1997-2015, and we have shown a progressive increase in the odds of developing hyperkalaemia and acute kidney injury after a UTI in more recent years. The increase in hyperkalaemia may be due to an increased rate of blood testing in primary care (particularly among groups at risk of high potassium levels, such as patients with diabetes or chronic kidney disease) or improved automatic recording of test results in general practice records. The marked increase in acute kidney injury over time as defined by Hospital Episode Statistics (HES) coding is well established and likely to be predominantly related to increased clinical focus and the adoption of consensus definitions defined by changes in creatinine levels.^34^ ^35^ By adjusting for calendar period we aimed to minimise the influence of these secular trends on our results.

In contrast to previous studies, we did not identify an increased risk of death from any cause in users of trimethoprim. The two previous papers that identified an increased risk of sudden death among users of renin-angiotensin system blockade taking co-trimoxazole, used a case-control design with cases defined by sudden death, among residents of Ontario over 18 years of follow-up.^2^ ^21^ They were therefore well powered for this outcome. We chose all cause death as a prespecified analysis owing to lack of power for cause specific death, since we restricted the cohort to patients with a UTI to address issues of confounding by indication for antibiotic choice that had limited previous research. In addition, since our cohort was not restricted to users of renin-angiotensin system blockers, the overall risk of sudden death was likely to be lower in our study. However, acknowledging these limitations, our findings of an odds ratio of death (comparing trimethoprim with amoxicillin) within seven days of a UTI of 1.51 (95% confidence interval 0.87 to 2.65) among users of renin-angiotensin system blockers are consistent with those of Fralick et al (adjusted odds ratio 1.38, 95% confidence interval 1.09 to 1.76).^2^ However, our study also had greater ability to adjust for detailed characteristics, such as estimated glomerular filtration rate before UTI, which are likely to have reduced residual confounding. While we cannot exclude a small increase in the odds of sudden death after trimethoprim use among users of renin-angiotensin system blockers, we have found no evidence of an association between trimethoprim use and death in the whole population of older adults, and sudden death is a rare outcome (1.1% (6 in 542) of total deaths within 14 days of antibiotic initiation among users of renin-angiotensin system blockers).

### Clinical implications

Recent national prescribing guidance recommends nitrofurantoin as the first line choice for treating UTIs in adults, with trimethoprim an equivalent choice for those with low risk of antimicrobial resistance, meaning that trimethoprim will continue to be commonly prescribed.^10^ Current British National Formulary prescribing guidelines for trimethoprim mention an unquantified risk of hyperkalaemia (but not acute kidney injury), and a possible increase in risk if given with angiotensin-converting-enzyme (ACE) inhibitors, angiotensin-receptor antagonists, and spironolactone.^36^ Given the different rates of outcomes within subgroups of the population, it is useful to consider absolute and relative risk together by calculating the excess number of events attributable to trimethoprim treatment, although our very strict definition of a UTI means that these estimates should be treated with caution. As an example, our results suggest that for 1000 UTI episodes treated with antibiotics in those aged 65 and over not taking renin-angiotensin system blockers, treatment with trimethoprim, instead of amoxicillin, would result in one additional case of hyperkalaemia and two of acute kidney injury. Among those taking either renin-angiotensin system blockers or potassium-sparing diuretics the figures would be very similar; two additional cases of both hyperkalaemia and acute kidney injury. However, treatment with both renin-angiotensin system blockers and potassium-sparing diuretics would result in 18 additional cases of hyperkalaemia and 11 of acute kidney injury.

A small increased absolute risk of a rare outcome (such as in the general population) from trimethoprim may be acceptable when set against a need for multiple treatment options for patients with allergy to other antibiotics or bacterial resistance patterns. While acute kidney injury and hyperkalaemia may result in avoidable morbidity and hospital admission, it is reassuring that we have not identified an increased risk of death, suggesting that there is appropriate response to these outcomes. Our results show that trimethoprim continues to be prescribed to people at high risk of adverse outcomes including patients with advanced renal impairment and those taking renin-angiotensin system blockers with potassium-sparing diuretics. For groups at high risk of acute kidney injury and hyperkalaemia, other antibiotics should be considered, but, if this is not possible, monitoring of renal function and potassium levels should be performed, in line with the existing summary of product characteristics for trimethoprim. 

### Conclusion

Our results show that trimethoprim is associated with greater risk of acute kidney injury and hyperkalaemia compared with other antibiotic drugs for a UTI, among the general population aged 65 and over, and not just those treated with renin-angiotensin system blockers. However, this is not associated with an increased risk of death.

**What is already known on this topic**

Co-trimoxazole (a combination antibiotic drug containing trimethoprim and sulfamethoxazole) has been associated with an increased risk of sudden death, which may be mediated by increased serum potassium
Previous research is limited to specific patient groups (eg, patients taking renin-angiotensin system blockers) and is limited by possible confounding by type and severity of infection
It is not known if the risks for trimethoprim are similar to those for co-trimoxazole

**What this study adds**

Compared with amoxicillin, the risk of acute kidney injury and hyperkalaemia increased in the two weeks after taking trimethoprim for a UTI
The risk of sudden death was not higher among patients prescribed trimethoprim compared with amoxicillin
Trimethoprim is associated with a greater risk of acute kidney injury and hyperkalaemia compared with other antibiotic drugs for a UTI among the general population as well as those taking renin-angiotensin system blockers

## Web Extra

Extra material supplied by the author

## Notes

Contributors: LAT had the original idea for the study. All authors were involved in the study design. EC and KEM contributed equally to this paper. EC undertook the data management and primary analysis, and wrote early drafts of the manuscript. KM supervised each stage of data management and preliminary analyses, and wrote the first complete manuscript draft. CL undertook the inverse probability of treatment weighting analysis. All authors contributed to further drafts and approved the final manuscript. All authors had full access to the data in the study. EC and KEM are the guarantors.

Ethical approval: This study was approved by the London School of Hygiene and Tropical Medicine Research Ethics Committee (reference 12014) and by the Clinical Practice Research Datalink independent scientific advisory committee (ISAC protocol number: 16_087R).

Data sharing: No additional data are available.

Transparency: The manuscripts guarantors (EC and KEM) affirm that this manuscript is an honest, accurate, and transparent account of the study being reported; that no important aspects of the study have been omitted; and that any discrepancies from the study as planned have been explained.
