# Supplementary material for: Trimethoprim use for urinary tract infection and risk of adverse outcomes in older patients: cohort study
Source: BMJ. 2018 Feb 9;360:k341. doi: 10.1136/bmj.k341 (PMC5806507; doi:10.1136/bmj.k341)
Supplement: Supplementary file 1 — Appendix: Supplementary materials [file cree040349.ww1.pdf]

SUPPLEMENTARY APPENDIX

Text S1: Inverse probability of treatment weighting analysis

To ensure that we were assessing risk of sudden death, acute kidney injury, and hyperkalaemia between comparable groups (to reduce confounding by indication), we examined the risks of all three outcomes between groups of trimethoprim and amoxicillin users after propensity score weighting (IPTW). We calculated propensity scores for patients using logistic regression to predict choice of antibiotic (trimethoprim or amoxicillin). Factors included in the propensity score model were the covariates adjusted for in the primary analysis (age, sex, renal function, chronic comorbidities, history of renal or urological disease, calendar period, and co-prescription with renin-angiotensin system blockers or potassium-sparing diuretics). Then, for each patient, a weight defined as the inverse of the probability the treatment they had received was calculated. Finally, a weighted logistic regression was used to estimate the marginal odds ratios (ORs) for each of the three outcomes.

The propensity score was first estimated with a logistic regression including all the variables listed in **Table 1** (n=273,736). When estimating the standardized differences before propensity score weighting, only age, sex, cardiac failure and arrhythmia were unbalanced (standardized difference >10%, see Table 1). After weighting on the propensity score, all variables were balanced (**Table 1**).

Table 1. Balance of the covariates before and after propensity score adjustment (n=273,736)

Variable	Standardised difference (%)	
	Before IPTW	After IPTW
**Age**	13.0	0.5
**Sex**	11.2	0.3
**Renal function**	5.7	0.5
**Comorbidities**		
Diabetes mellitus	6.1	0.7
Ischaemic heart disease	7.3	0.6
Cardiac failure	11.3	0.8
Arrhythmia	11.1	0.5
Hypertension	5.5	0.3
**History of renal/urological disease**		
Prostatic hypertrophy	4.7	0.4
Calculi	4.7	0.0
Malignancy	1.3	0.4
Urological structural abnormalities	3.7	0.2
**Co-prescription**		
RAS blocker	4.1	0.3
Potassium-sparing diuretic	4.4	0.4
**Other**		
Calendar period	0.5	0.0

RAS blocker: Angiotensin converting enzyme inhibitor/angiotensin receptor blocker
IPTW: inverse probability of treatment weighting

Results

Table 2 gives the results of the multivariable regression and inverse probability treatment weighting (IPTW) propensity score approaches for the three outcomes.

Table 2. Results comparing the odds of death, acute kidney injury, or hyperkalaemia in those prescribed trimethoprim for urinary tract infection compared to those prescribed amoxicillin using different analysis approaches.

	Acute kidney injury			Hyperkalaemia			Death		
**Method**	**n**	**OR**	**95% CI**	**n**	**OR**	**95% CI**	**n**	**OR**	**95% CI**
Multivariable regression	273,736	1.73	[1.34;2.24]	273,736	2.26	[1.51;3.38]	273,736	0.90	[0.76;1.07]
IPTW		1.65	[1.27;2.14]		2.48	[1.64;3.75]		0.85	[0.72;1.01]

IPTW: inverse probability treatment weighting.

Note that multivariable regression and IPTW give different estimates because the former aims to estimate conditional effects and the latter marginal effects

Table S1. Odds ratios (95% CIs) for AKI, hyperkalaemia and death within 14 days of antibiotic treatment for urinary tract infection in sequentially adjusted logistic regression models.

		**Acute kidney injury odds ratio (95% CI)**			**Hyperkalaemia odds ratio (95% CI)**			**Death odds ratio (95% CI)**		
		Adjusted for age and sex	Adjusted for age, sex, calendar year and comorbidities	Fully adjusted	Adjusted for age and sex	Adjusted for calendar year and comorbidities	Fully adjusted	Adjusted for age and sex	Adjusted for calendar year and comorbidities	Fully adjusted
		n=422,514	n=422,514	n=422,514	n=422,514	n=422,514	n=422,514	n=422,514	n=422,514	n=422,514
**Class of antibiotic (exposure of interest)**	Amoxicillin	reference	reference	reference	reference	reference	reference	reference	reference	reference
	Trimethoprim	1.43 (1.09-1.87)	1.49 (1.13-1.95)	1.72 (1.31-2.24)	1.82 (1.19-2.78)	1.93 (1.26-2.95)	2.27 (1.49-3.45)	0.86 (0.72-1.02)	0.88 (0.74-1.04)	0.90 (0.76-1.07)
	Cefalexin	0.70 (0.51-0.96)	0.97 (0.71-1.31)	1.01 (0.74-1.37)	1.01 (0.65-1.57)	1.12 (0.72-1.74)	1.20 (0.77-1.85)	0.97 (0.80-1.18)	0.96 (0.79-1.17)	0.97 (0.80-1.18)
	Ciprofloxacin	1.18 (0.82-1.69)	1.41 (0.98-2.02)	1.48 (1.03-2.13)	1.05 (0.60-1.83)	1.12 (0.64-1.96)	1.17 (0.68-2.02)	0.90 (0.72-1.12)	0.90 (0.72-1.13)	0.92 (0.73-1.15)
	Nitrofurantoin	1.05 (0.76-1.45)	0.73 (0.52-1.01)	0.89 (0.65-1.24)	1.04 (0.63-1.70)	0.87 (0.53-1.44)	1.07 (0.65-1.75)	0.66 (0.53-0.81)	0.69 (0.56-0.86)	0.72 (0.58-0.89)
**Sex**	Female	0.32 (0.28-0.36)	0.36 (0.32-0.41)	0.34 (0.29-0.39)	0.41 (0.34-0.49)	0.47 (0.39-0.56)	0.42 (0.34-0.51)	0.47 (0.43-0.51)	0.50 (0.46-0.54)	0.44 (0.40-0.49)
**Age**	65-69	reference	reference	reference	reference	reference	reference	reference	reference	reference
	70-74	1.41 (1.09-1.81)	1.35 (1.05-1.74)	1.22 (0.94-1.58)	1.06 (0.77-1.44)	0.96 (0.71-1.32)	0.82 (0.60-1.13)	1.58 (1.24-2.02)	1.56 (1.22-2.00)	1.57 (1.23-2.01)
	75-79	1.92 (1.51-2.44)	1.69 (1.33-2.15)	1.34 (1.04-1.71)	1.32 (1.01-1.72)	1.11 (0.85-1.46)	0.83 (0.63-1.10)	2.41 (1.97-2.94)	2.30 (1.88-2.81)	2.30 (1.87-2.82)
	80-84	3.23 (2.55-4.07)	2.55 (2.01-3.23)	1.78 (1.38-2.29)	1.96 (1.49-2.59)	1.51 (1.15-1.98)	0.98 (0.74-1.30)	4.12 (3.34-5.09)	3.77 (3.04-4.67)	3.68 (2.96-4.57)
	85+	6.08 (4.92-7.53)	4.18 (3.36-5.21)	2.47 (1.96-3.12)	2.28 (1.73-3.01)	1.62 (1.22-2.14)	0.91 (0.68-1.23)	10.91 (8.90-13.36)	9.29 (7.52-11.47)	8.52 (6.89-10.54)
**Calendar period**	1997-2000		reference	reference		reference	reference		reference	reference
	2001-2004		1.72 (1.03-2.87)	1.62 (0.96-2.74)		2.24 (1.35-3.71)	1.81 (1.08-3.03)		0.92 (0.79-1.07)	0.98 (0.83-1.15)
	2005-2008		3.90 (2.42-6.28)	3.67 (2.20-6.12)		2.93 (1.87-4.59)	2.18 (1.32-3.60)		0.92 (0.80-1.06)	1.03 (0.86-1.23)
	2009-2011		7.75 (4.87-12.35)	8.01 (4.85-13.22)		4.07 (2.56-6.47)	3.34 (2.00-5.59)		0.82 (0.69-0.97)	0.94 (0.78-1.13)
	2012-2015		13.70 (8.87-21.17)	15.22 (9.47-24.45)		4.08 (2.51-6.63)	3.67 (2.18-6.18)		0.93 (0.78-1.10)	1.07 (0.87-1.31)
**Comorbidity**	Diabetes mellitus		1.57 (1.41-1.74)	1.37 (1.23-1.52)		2.36 (1.98-2.82)	1.82 (1.52-2.19)		1.12 (1.00-1.25)	1.12 (1.00-1.26)
	IHD		0.90 (0.80-1.01)	0.86 (0.76-0.96)		1.07 (0.89-1.28)	0.94 (0.78-1.13)		0.95 (0.86-1.04)	0.97 (0.87-1.07)
	Cardiac failure		2.30 (2.01-2.62)	1.65 (1.44-1.90)		2.12 (1.71-2.63)	1.20 (0.97-1.47)		2.06 (1.82-2.33)	2.00 (1.77-2.27)
	Arrhythmia		1.37 (1.20-1.55)	1.32 (1.16-1.51)		1.12 (0.92-1.36)	1.03 (0.85-1.25)		1.24 (1.10-1.39)	1.25 (1.11-1.40)
	Hypertension		1.46 (1.28-1.67)	1.27 (1.10-1.47)		1.56 (1.27-1.92)	1.00 (0.80-1.25)		0.76 (0.70-0.84)	0.82 (0.74-0.90)
**Renal function (eGFR)**	eGFR <30			9.52 (7.75-11.68)			14.39 (11.06-18.74)			2.20 (1.84-2.64)
	eGFR 30-44			3.70 (3.08-4.43)			4.37 (3.39-5.65)			1.27 (1.10-1.45)
	eGFR 45-59			1.95 (1.65-2.30)			1.86 (1.45-2.40)			0.98 (0.84-1.13)
	eGFR >=60			reference			reference			reference
	Absent			1.93 (1.41-2.64)			1.45 (0.94-2.24)			1.30 (1.10-1.54)
**History of renal/urological disease**	Prostate			0.79 (0.65-0.96)			0.74 (0.53-1.03)			0.60 (0.49-0.73)
	Calculi			0.90 (0.66-1.22)			0.97 (0.61-1.53)			0.78 (0.57-1.08)
	Malignancy			1.11 (0.43-2.89)			0.99 (0.24-4.10)			1.00 (0.37-2.70)
	Structural			1.13 (0.76-1.69)			0.87 (0.43-1.76)			0.44 (0.24-0.81)
**RAS blocker and/or potassium-sparing diuretic**	Neither			Reference			reference			reference
	RAS blocker *or* KSD			1.03 (0.92-1.16)			1.98 (1.60-2.44)			0.77 (0.69-0.85)
	RAS blocker *and* KSD			2.08 (1.63-2.67)			6.17 (4.54-8.39)			0.99 (0.76-1.29)

All models use robust standard errors to account for clustering by general practice.

UTI: urinary tract infection; IHD: Ischaemic heart disease; RAS blocker: renin-angiotensin system blocker; KSD: potassium-sparing diuretic; eGFR: estimated glomerular filtration rate; N/A: not applicable

Table S2. Adjusted odds ratios (95% CIs) for AKI, hyperkalaemia and death following antibiotic treatment for urinary tract infection in the main analysis and additional sensitivity analyses.

		N	Fully adjusted* odds ratio (95% CI)				
			Amoxicillin	Trimethoprim	Cefalexin	Ciprofloxacin	Nitrofurantoin
**Acute kidney injury**	**Main analysis***	**422,514**	**Reference**	**1.72 (1.31-2.24)**	**1.01 (0.74-1.37)**	**1.48 (1.03-2.13)**	**0.89 (0.65-1.24)**
	1. Using robust standard errors to account for clustering by patient as a result of patients contributing more than one UTI to the analysis.	422,514	Reference	1.72 (1.33-2.22)	1.01 (0.75-1.37)	1.48 (1.06-2.07)	0.89 (0.67-1.20)
	2. Antibiotic exposure defined as antibiotic prescription on the same day as a record of a UTI morbidity code.	416,680	Reference	1.80 (1.35-2.39)	1.03 (0.74-1.43)	1.55 (1.06-2.26)	0.91 (0.65-1.28)
	3. Excluding prescriptions for antibiotics where patient received antibiotic in the 28 days prior to UTI.	382,576	Reference	1.73 (1.30-2.29)	1.09 (0.79-1.49)	1.48 (1.01-2.17)	0.84 (0.59-1.20)
	4. Limiting the analysis to the first recorded antibiotic-treated UTI for each individual during eligible follow up.	126,459	Reference	1.58 (1.07-2.33)	0.85 (0.53-1.36)	1.26 (0.74-2.16)	0.85 (0.53-1.37)
	5. Antibiotic exposure defined as antibiotic prescription for any indication.	2,764,740	Reference	2.36 (2.22-2.51)	1.27 (1.16-1.40)	1.82 (1.65-2.02)	1.34 (1.21-1.47)
	6. AKI defined as occurring within 7 days after antibiotic prescription.	422,514	Reference	1.74 (1.24-2.44)	1.09 (0.74-1.59)	1.65 (1.08-2.53)	0.86 (0.59-1.26)
	7. Additionally adjusted for IMD quintile and lifestyle covariates: BMI, smoking status and alcohol status.	368,256	Reference	1.71 (1.30-2.27)	1.02 (0.73-1.43)	1.43 (0.98-2.09)	0.87 (0.62-1.21)
	8. Limiting study population to individuals with ethnicity recorded in CPRD or HES, who became eligible for study entry from 2006.**	189,574	Reference	1.76 (1.25-2.47)	0.94 (0.62-1.41)	1.43 (0.91-2.26)	0.89 (0.59-1.34)
	9. Limiting study population to users of RAS blockers on date of antibiotic prescription***	135,152	Reference	1.92 (1.29-2.87)	1.02 (0.63-1.63)	1.34 (0.76-2.38)	1.00 (0.64-1.55)
	10. Limiting to those with known baseline renal function.	343,166	Reference	1.72 (1.31-2.25)	1.03 (0.76-1.41)	1.45 (0.99-2.12)	0.86 (0.62-1.20)
**Hyperkalaemia**	**Main analysis***	**422,514**	**Reference**	**2.27 (1.49-3.45)**	**1.20 (0.77-1.85)**	**1.17 (0.68-2.02)**	**1.07 (0.65-1.75)**
	1. Using robust standard errors to account for clustering by patient as a result of patients contributing more than one UTI to the analysis.	422,514	Reference	2.27 (1.51-3.39)	1.20 (0.76-1.90)	1.17 (0.68-2.01)	1.07 (0.67-1.70)
	2. Antibiotic exposure defined as antibiotic prescription on the same day as a record of a UTI morbidity code.	416,680	Reference	2.30 (1.49-3.54)	1.21 (0.77-1.89)	1.18 (0.67-2.06)	1.03 (0.62-1.71)
	3. Excluding prescriptions for antibiotics where patient received antibiotic in the 28 days prior to UTI.	382,576	Reference	1.96 (1.30-2.95)	1.08 (0.70-1.66)	1.15 (0.67-1.98)	0.92 (0.56-1.51)
	4. Limiting the analysis to the first recorded antibiotic-treated UTI for each individual during eligible follow up.	126,459	Reference	1.18 (0.73-1.90)	0.48 (0.26-0.91)	0.79 (0.38-1.65)	0.64 (0.31-1.32)
	5. Antibiotic exposure defined as antibiotic prescription for any indication.	2,764,740	Reference	2.52 (2.30-2.76)	1.29 (1.14-1.46)	1.42 (1.23-1.64)	1.26 (1.10-1.44)
	6. Hyperkalaemia defined as occurring within 7 days after antibiotic prescription.	422,514	Reference	2.16 (1.27-3.68)	1.28 (0.73-2.25)	1.13 (0.57-2.23)	0.84 (0.44-1.61)
	7. Additionally adjusted for IMD quintile and lifestyle covariates: BMI, smoking status and alcohol status.	368,256	Reference	2.25 (1.45-3.48)	1.26 (0.79-2.02)	1.19 (0.66-2.14)	0.97 (0.58-1.64)
	8. Limiting study population to individuals with ethnicity recorded in CPRD or HES, who became eligible for study entry from 2006.**	189,574	Reference	2.01 (1.21-3.33)	0.78 (0.42-1.46)	0.76 (0.34-1.70)	0.92 (0.50-1.67)
	9. Limiting study population to users of RAS blockers on date of antibiotic prescription***	135,152	Reference	2.22 (1.34-3.68)	0.87 (0.50-1.51)	1.12 (0.56-2.26)	0.91 (0.49-1.68)
	10. Limiting to those with known baseline renal function.	343,166	Reference	2.25 (1.45-3.47)	1.19 (0.76-1.87)	1.22 (0.70-2.14)	1.07 (0.65-1.78)
**Death**	**Main analysis***	**422,514**	**Reference**	**0.90 (0.76-1.07)**	**0.97 (0.80-1.18)**	**0.92 (0.73-1.15)**	**0.72 (0.58-0.89)**
	1. Using robust standard errors to account for clustering by patient as a result of patients contributing more than one UTI to the analysis.	422,514	Reference	0.90 (0.76-1.07)	0.97 (0.80-1.18)	0.92 (0.72-1.16)	0.72 (0.59-0.89)
	2. Antibiotic exposure defined as antibiotic prescription on the same day as a record of a UTI morbidity code.	416,680	Reference	0.94 (0.79-1.12)	1.01 (0.82-1.24)	0.95 (0.75-1.20)	0.73 (0.59-0.91)
	3. Excluding prescriptions for antibiotics where patient received antibiotic in the 28 days prior to UTI.	382,576	Reference	0.86 (0.73-1.03)	0.98 (0.80-1.19)	0.85 (0.66-1.09)	0.73 (0.58-0.91)
	4. Limiting the analysis to the first recorded antibiotic-treated UTI for each individual during eligible follow up.	126,459	Reference	0.88 (0.67-1.15)	1.06 (0.77-1.45)	0.77 (0.49-1.19)	0.83 (0.59-1.15)
	5. Antibiotic exposure defined as antibiotic prescription for any indication.	2,764,740	Reference	0.71 (0.68-0.75)	0.83 (0.78-0.88)	0.87 (0.81-0.94)	0.51 (0.47-0.55)
	6. Sudden death defined as occurring within 7 days after antibiotic prescription.	422,514	Reference	0.89 (0.72-1.12)	0.94 (0.72-1.21)	0.83 (0.59-1.15)	0.63 (0.48-0.84)
	7. Additionally adjusted for IMD quintile and lifestyle covariates: BMI, smoking status and alcohol status.	368,256	Reference	0.81 (0.67-0.98)	0.87 (0.69-1.09)	0.83 (0.63-1.10)	0.72 (0.57-0.92)
	8. Limiting study population to individuals with ethnicity recorded in CPRD or HES, who became eligible for study entry from 2006.**	189,574	Reference	1.20 (0.88-1.63)	1.38 (0.99-1.93)	1.06 (0.70-1.62)	0.92 (0.66-1.29)
	9. Limiting study population to users of RAS blockers on date of antibiotic prescription and death within 7 days***	135,152	Reference	1.51 (0.87-2.65)	1.25 (0.65-2.42)	1.00 (0.52-1.93)	0.94 (0.49-1.80)
	10. Limiting study population to users of RAS blockers on date of antibiotic prescription and death within 14 days***	135,152	Reference	1.12 (0.80-1.57)	0.94 (0.61-1.44)	1.05 (0.66-1.67)	0.85 (0.57-1.29)
	11. Limiting to those with known baseline renal function	343,166	Reference	0.95 (0.79-1.14)	0.97 (0.78-1.14)	0.91 (0.71-1.16)	0.77 (0.61-0.97)

*Unless otherwise stated, adjusted for the following recorded on or before the data of antibiotic prescription: age, sex, chronic comorbidities, calendar period, renal or urological disease, baseline renal function, and use of RAS blockers and/or potassium-sparing diuretics. **Using ethnicity both in the equation used to calculate eGFR and as covariate in the analysis. ***Adjusted for use of potassium-sparing diuretics. IMD: Index of multiple deprivation; BMI: body mass index; UTI: urinary tract infection
